# Supplementary material for: Genomic Investigation of Salmonella Typhi in Hong Kong Revealing the Predominance of Genotype 3.2.2 and the First Case of an Extensively Drug-Resistant H58 Genotype
Source: Microorganisms. 2023 Mar 6;11(3):667. doi: 10.3390/microorganisms11030667 (PMC10058776; doi:10.3390/microorganisms11030667)

Supplementary file S1

Figure S1. **Phylogenetic analysis of 569 *Salmonella* Typhi strains of genotype 4.3.1.1.P1.** A mid-point rooting maximum likelihood phylogenetic tree of 568 *Salmonella* Typhi isolates from the GenBank and strain S3 (indicated by the red branch and using red font color) from this study. Except for S3, all the isolates were originated from Pakistan, 2016-2018.

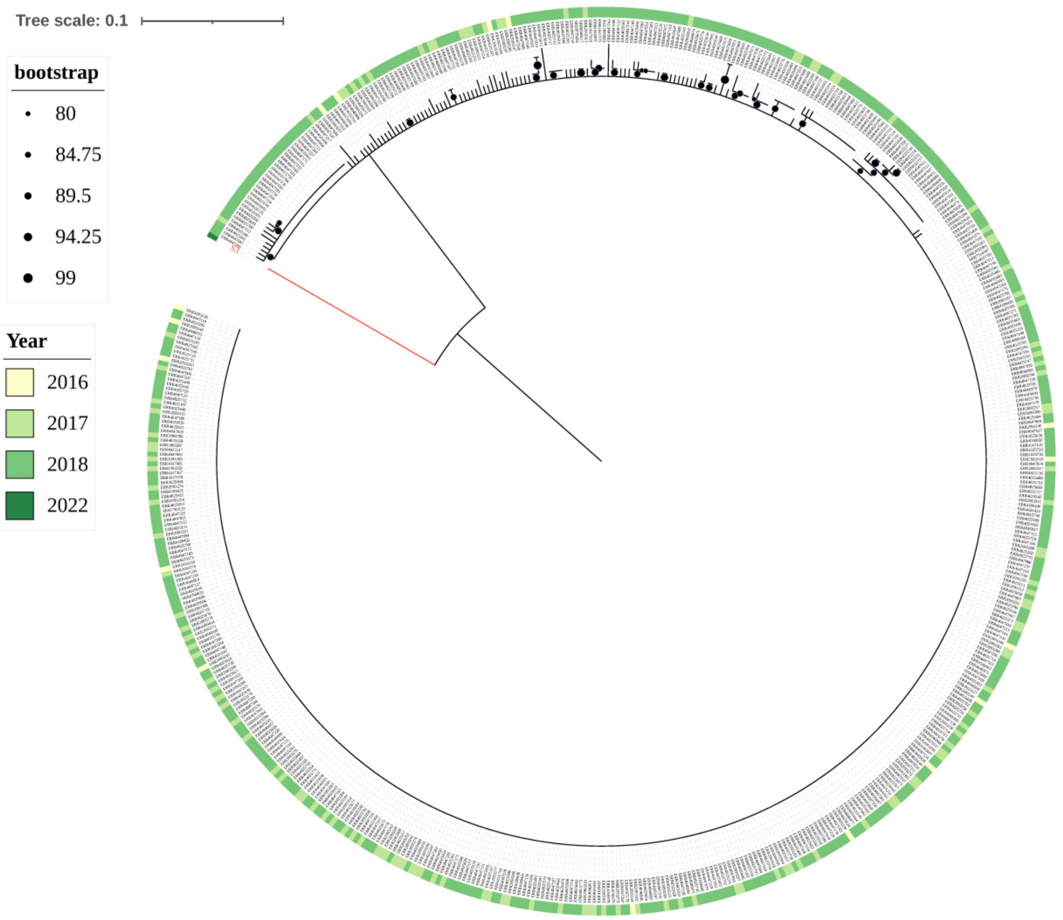

Figure S2. **Phylogenetic analysis of 17 *Salmonella* Typhi strains of genotype 2.3.4.** A mid-point rooting maximum likelihood phylogenetic tree of 17 *Salmonella* Typhi isolates from the GenBank and strain S7 (red font color) from this study.

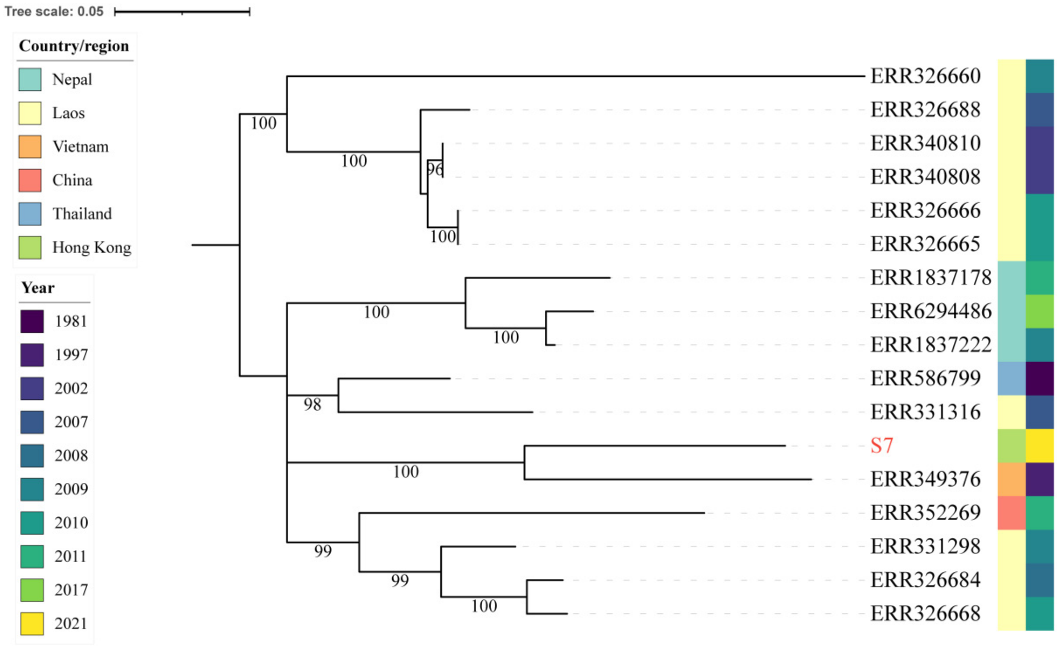

Supplement: Supplementary file 1 [file microorganisms-11-00667-s001.zip › microorganisms-2220969-supplementary-Figures S1 and S2.pdf]
